# Accurate and Automated Genotyping of the *CFTR* Poly-T/TG Tract with *CFTR*-TIPS

**DOI:** 10.3390/ijms25158533

**Published:** 2024-08-05

**Authors:** Qiliang Ding, Christopher D. Hofich, Tifani B. Kellogg, Rhonda K. Kuennen, Kaitlin N. Paxton, Sarah M. Thieke, Kandelaria M. Rumilla, Linda Hasadsri

**Affiliations:** Division of Laboratory Genetics and Genomics, Department of Laboratory Medicine and Pathology, Mayo Clinic, Rochester, MN 55905, USA

**Keywords:** *CFTR*, cystic fibrosis, poly-T tract, Sanger sequencing, molecular diagnostics

## Abstract

Cystic fibrosis is caused by biallelic pathogenic variants in the *CFTR* gene, which contains a polymorphic (TG)_m_T_n_ sequence (the “poly-T/TG tract”) in intron 9. While T_9_ and T_7_ alleles are benign, T_5_ alleles with longer TG repeats, e.g., (TG)_12_T_5_ and (TG)_13_T_5_, are clinically significant. Thus, professional medical societies currently recommend reporting the TG repeat size when T_5_ is detected. Sanger sequencing is a cost-effective method of genotyping the (TG)_m_T_n_ tract; however, its polymorphic length substantially complicates data analysis. We developed *CFTR*-TIPS, a freely available web-based software tool that infers the (TG)_m_T_n_ genotype from Sanger sequencing data. This tool detects the (TG)_m_T_n_ tract in the chromatograms, quantifies goodness of fit with expected patterns, and visualizes the results in a graphical user interface. It is broadly compatible with any Sanger chromatogram that contains the (TG)_m_T_n_ tract ± 15 bp. We evaluated *CFTR*-TIPS using 835 clinical samples previously analyzed in a CLIA-certified, CAP-accredited laboratory. When operated fully automatically, *CFTR*-TIPS achieved 99.8% concordance with our clinically validated manual workflow, while generally taking less than 10 s per sample. There were two discordant samples: one due to a co-occurring heterozygous duplication that confounded the tool and the other due to incomplete (TG)_m_T_n_ tract detection in the reverse chromatogram. No clinically significant misclassifications were observed. *CFTR*-TIPS is a free, accurate, and rapid tool for CFTR (TG)_m_T_n_ tract genotyping using cost-effective Sanger sequencing. This tool is suitable both for automated use and as an aid to manual review to enhance accuracy and reduce analysis time.

## 1. Introduction

Cystic fibrosis (CF) is one of the most common genetic diseases, impacting an estimated 160,000 living patients worldwide [1]. As an autosomal recessive condition, CF is caused by biallelic (homozygous or compound heterozygous) pathogenic variants in the *CFTR* gene. *CFTR* encodes a transmembrane chloride transporter, and its dysfunction leads to altered secretions, obstruction, and/or destruction in multiple organs (e.g., lungs, pancreas, and intestine) [2].

Despite significant advancements in therapies for CF patients, the life expectancy of those affected with the most severe form of the disease, also known as “classic CF”, is less than 50 years [3], with respiratory failure being the leading cause of mortality [4]. In addition to “classic CF”, pathogenic variants in the *CFTR* gene may also cause less severe *CFTR*-related disorders, such as *CFTR*-related pancreatitis [5] and congenital bilateral absence of the vas deferens (CBAVD) [6].

In the United States, approximately one in thirty-five individuals carries at least one pathogenic variant in *CFTR*. These carriers are at risk of having a child with CF if their reproductive partner is also a carrier. Ashkenazi Jewish and European Americans are more likely to be CF carriers, with an estimated frequency of one in twenty-five individuals [7]. Because of the significant carrier rate and the high disease severity, the American College of Obstetricians and Gynecologists (ACOG) recommends that CF carrier screening be offered to all women who are pregnant or considering pregnancy [8].

**Figure 1:**
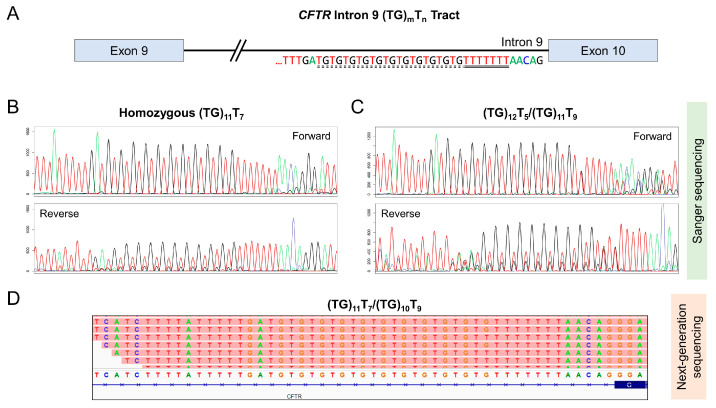
Analysis of the *CFTR* (TG)_m_T_n_ tract using Sanger and next-generation sequencing. (**A**) Overview of the (TG)_m_T_n_ tract in *CFTR* intron 9. Dashed underline: the TG dinucleotide repeat. Solid underline: the poly-T repeat. (**B**,**C**) Bidirectional Sanger chromatograms for a sample homozygous for the (TG)_11_T_7_ allele (**B**) or compound heterozygous for the (TG)_12_T_5_ and (TG)_11_T_9_ alleles (**C**). In (**C**), the different lengths of (TG)_12_T_5_ (29 bp) and (TG)_11_T_9_ (31 bp) caused overlapping peaks in the Sanger chromatograms, complicating interpretation. (**D**) NGS reads, visualized using the Integrative Genomic Viewer (IGV) [20], for a sample compound heterozygous for the (TG)_11_T_7_ and (TG)_10_T_9_ alleles. The genotype could be readily resolved using individual reads.

In clinical laboratories, *CFTR* sequence analysis is complicated by a region with low sequence complexity in intron 9 of the gene. This region contains a TG dinucleotide repeat followed by a poly-T mononucleotide repeat, hereafter referred to as the (TG)_m_T_n_ tract (Figure 1A). Both (TG)_m_ and T_n_ are polymorphic, with most individuals carrying T_7_, or “7T” (Figure 1B). While less common, T_5_ and T_9_ alleles, also known as “5T” and “9T”, have been reported (Figure 1C,D) [9].

The (TG)_m_T_n_ tract is in the splice acceptor region of intron 9 [10] and is responsible for the proper inclusion of exon 10 in the mature mRNA [11]. Exon 10 is required for a functional CFTR protein. The T_9_ and T_7_ alleles (with any TG repeat size) are clinically benign, as is the (TG)_11_T_5_ allele [12]. On the other hand, T_5_, in combination with longer TG repeats, such as (TG)_12_T_5_ and (TG)_13_T_5_, is clinically significant due to substantial exon 10 mis-splicing [9,13]. These alleles are enriched in CBAVD patients [14]. They also act as genetic modifiers that increase the severity and penetrance of the *CFTR* R117H variant, which, by itself, is a mild and low-penetrance pathogenic variant, in causing classic CF [15]. Moreover, they have been reported to cause classic CF when present in *trans* with another severe pathogenic variant such as *CFTR* F508del (a.k.a., ΔF508).

Because only (TG)_12_T_5_ and (TG)_13_T_5_ are considered clinically significant, while (TG)_11_T_5_ is not, the American College of Medical Genetics and Genomics (ACMG) recently recommended that molecular testing laboratories determine and report the TG repeat size whenever T_5_ is detected [16,17]. Clinical assays largely use one of two technical approaches to determine the TG repeat size: Sanger sequencing [18] and targeted next-generation sequencing (NGS) [9,19]. Although Sanger sequencing is less expensive and has a faster turnaround time, compound heterozygosity (i.e., individuals with two different (TG)_m_T_n_ alleles) in this low-complexity region complicates review of the Sanger chromatogram (Figure 1C). In our clinically validated workflow, manual interpretation by an experienced technologist is required to resolve the genotypes. In contrast, sequencing reads from NGS can readily resolve the (TG)_m_T_n_ allele genotypes (Figure 1D); however, its higher cost limits widespread application in cost-conscious settings.

Here, we present *CFTR*-TIPS (*CFTR* Tool for Inferring Poly-T/TG Size) version 1.0, a web-based software tool that automates the inference of the *CFTR* (TG)_m_T_n_ genotype from bidirectional Sanger chromatograms. This software is compatible with any Sanger chromatogram that contains the (TG)_m_T_n_ tract ± 15 bp. We evaluated *CFTR*-TIPS using 835 samples previously tested for the (TG)_m_T_n_ tract in a Clinical Laboratory Improvement Amendment (CLIA)-certified, College of American Pathologists (CAP)-accredited clinical laboratory. *CFTR*-TIPS achieved 99.8% concordance with the clinically validated manual workflow, and there were no clinically significant misclassifications. 

*CFTR*-TIPS enables efficient and accurate inference of the *CFTR* (TG)_m_T_n_ tract genotype using cost-effective Sanger sequencing. A preview version of *CFTR*-TIPS can be found at https://qd29.shinyapps.io/cftr-tips/ (accessed on 2 August 2024). Source code is available at https://github.com/qd29/cftr-tips/ (accessed on 2 August 2024) for local implementations.

## 2. Results

### 2.1. Architecture of CFTR-TIPS

**Figure 2:**
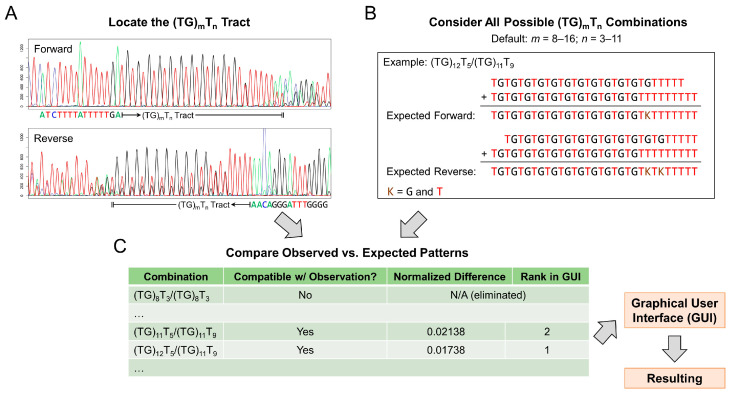
Architecture of *CFTR*-TIPS. (**A**) *CFTR*-TIPS scans the chromatograms to locate the (TG)_m_T_n_ tract based on flanking sequences. (**B**) *CFTR*-TIPS generates the expected peak patterns for all possible (TG)_m_T_n_ genotypes in the user-defined search space. The two alleles (possibly of different lengths) are aligned left (5′-) in the forward direction and aligned right (3′-) in the reverse direction. (**C**) *CFTR*-TIPS compares the observed and expected patterns. Genotypes incompatible with the observed peak pattern are eliminated. The remaining genotypes are visualized in a GUI, sorted by goodness of fit. A lower normalized difference score denotes better goodness of fit.

The input of *CFTR*-TIPS consists of bidirectional (forward and reverse) Sanger chromatogram (.ab1) files for a given sample in the ABIF format. These files are generated by Applied Biosystems DNA analyzers (Waltham, MA, USA). *CFTR*-TIPS outputs potential (TG)_m_T_n_ allele combinations (i.e., genotypes) that may match the input chromatograms, ranks them by goodness of fit, and visualizes their expected peak patterns alongside the observed chromatograms. The visualizations assist the user in determining the most likely (TG)_m_T_n_ genotype in the sample. The architecture of *CFTR*-TIPS is described in detail below and illustrated in Figure 2.

*CFTR*-TIPS first scans the input chromatograms to locate the (TG)_m_T_n_ tract. In the forward chromatogram (Figure 2A, upper panel), the 5′-most position is anchored using the upstream 15 bp flanking sequence, and the tract ends when the signal from thymine is no longer detected. Similarly, in the reverse chromatogram (Figure 2A, lower panel), the 3′-most position is anchored using the downstream 15 bp flanking sequence, and the tract ends when the signal from adenine is first detected. The end positions may be inaccurate due to potential peak overlaps; nonetheless, this has been factored into *CFTR*-TIPS. Overall, this approach allows *CFTR*-TIPS to be broadly compatible with any primer design that sequences the (TG)_m_T_n_ tract ± 15 bp.

*CFTR*-TIPS then exhaustively generates the expected peak patterns for all possible (TG)_m_T_n_ genotypes in a user-defined search space (Figure 2B). The default space encompasses T_3_ to T_11_ in combination with (TG)_8_ to (TG)_16_, encompassing all known (TG)_m_T_n_ alleles.

**Figure 3:**
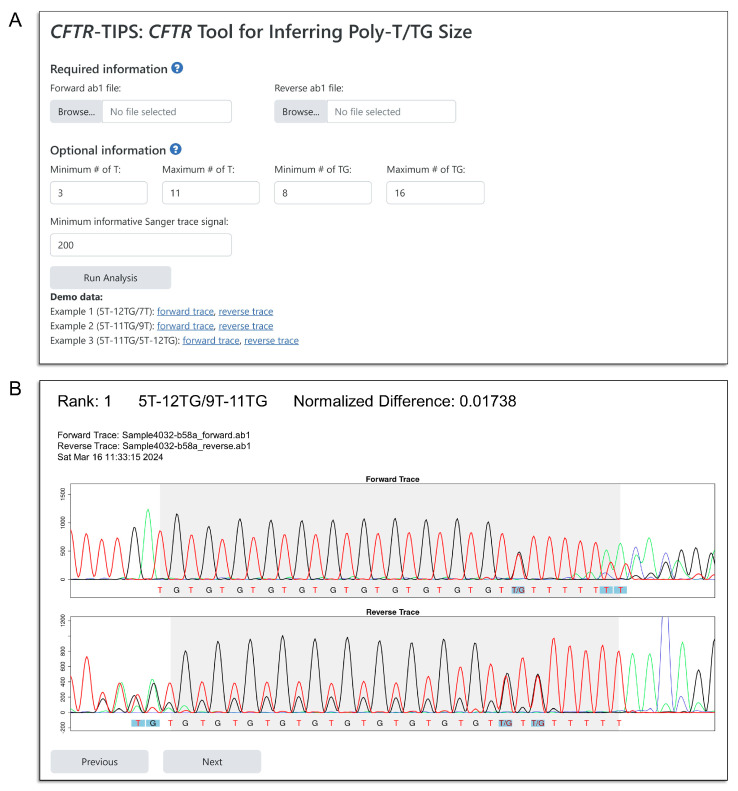
Graphical user interface of *CFTR*-TIPS. (**A**) The user input section of the *CFTR*-TIPS GUI. The forward and reverse Sanger chromatograms (.ab1 files) are required. The user may adjust additional parameters in the “Optional information” section. (**B**) The output section of the *CFTR*-TIPS GUI. *CFTR*-TIPS plots the *observed* chromatograms (as colored peaks) alongside the *expected* peak pattern of a given (TG)_m_T_n_ genotype (as T, G, or T/G letters under the peaks). By default, the genotype with the best fit is displayed. The letters in the shaded blue boxes denote positions at which the expected nucleotide(s) differ among the possible genotypes (compare with Figure 4). The gray shaded areas in the figure denote the detected (TG)_m_T_n_ tract. In the reverse chromatogram, the guanine (black) signals at positions at which only thymine (red) is expected represent signal bleed-through.

*CFTR*-TIPS next compares the observed and expected peak patterns. Genotypes are eliminated if their expected patterns are incompatible with the observation (e.g., at a given position, a specific nucleotide is expected, but its signal not observed). For the remaining genotypes, *CFTR*-TIPS calculates their goodness of fit (using a normalized difference score, see Methods) with the observed peak pattern (Figure 2C). Sorted by goodness of fit, the expected patterns for these genotypes are visualized in the graphical user interface (GUI), alongside the observed Sanger chromatograms (Figure 3).

### 2.2. Graphical User Interface of CFTR-TIPS

The web-based GUI is divided into user input and output sections. Example data from three de-identified samples are also provided in the GUI.

For the user input section (Figure 3A), the forward and reverse Sanger chromatograms, using the .ab1 file extension, are required. The user may optionally re-define the search space of (TG)_m_T_n_ alleles (using the “Minimum # of T”, “Maximum # of T”, “Minimum # of TG”, and “Maximum # of TG” parameters in the “Optional information” section). Given that the default search space encompasses all known (TG)_m_T_n_ alleles, adjustments to these parameters will rarely, if at all, be necessary. The user may also optionally adjust the “Minimum informative Sanger trace signal” parameter. In the chromatograms, positions with signal intensity below this value will be ignored when comparing the observed and expected patterns. We recommend adjusting this parameter based on the overall quality of the user’s Sanger chromatograms.

After the user clicks the “Run Analysis” button, the output section (Figure 3B) visualizes the *observed* peak pattern of the (TG)_m_T_n_ tract in the uploaded chromatograms, alongside the *expected* pattern for a given (TG)_m_T_n_ genotype (shown as letters, i.e., T, G, or T/G, below the observed chromatograms, see Figure 3B). The top of the image displays the name of the genotype, its rank among all possible genotypes, its normalized difference score, and additional metadata. By default, the genotype with the best fit (i.e., lowest normalized difference score) is displayed. The user may navigate among all possible genotype using the “Previous” and “Next” buttons at the bottom of the page.

**Figure 4:**
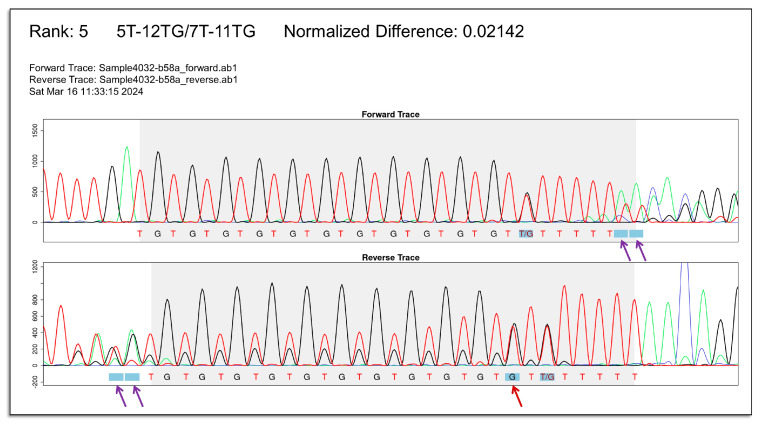
*CFTR*-TIPS facilitates comparison between observed and expected peak patterns. The observed Sanger chromatograms of the same sample as in Figure 3B are plotted. In this figure, the expected peak pattern of the fifth-ranked genotype (TG)_12_T_5_/(TG)_11_T_7_ is plotted. Mismatches between observed and expected peak patterns were observed at five of the seven shaded positions (red and purple arrows), including four positions at which thymine and/or guanine peak(s) were observed but not expected (purple arrows). In Figure 3B, except for one position complicated by overlapping peaks, the observed and expected peak patterns matched at six other positions. The comparison between Figure 3B and Figure 4 supports the interpretation that the (TG)_12_T_5_/(TG)_11_T_9_ genotype better explains the observed Sanger chromatograms in this sample.

In the image, some positions in the expected peak pattern may be shaded in blue (Figure 3B). At these positions, the expected nucleotide(s) differ among the possible genotypes. Thus, they are highly informative in determining the most likely (TG)_m_T_n_ genotype in a given sample. For example, Figure 3B shows the (TG)_12_T_5_/(TG)_11_T_9_ genotype, ranked first for the uploaded Sanger chromatograms. Except for the 5′-most shaded position in the reverse chromatogram (complicated by overlapping peaks), the observed and expected patterns matched at six other shaded positions. In contrast, Figure 4 shows the (TG)_12_T_5_/(TG)_11_T_7_ genotype for the same uploaded chromatograms, which was ranked fifth. In this image, the observed and expected patterns showed mismatches at five of the seven shaded positions, including four positions (two each in the forward and reverse chromatograms) at which peak(s) from thymine and/or guanine were observed but not expected due to the shorter expected tract length for (TG)_12_T_5_/(TG)_11_T_7_. Based on Figure 3B and Figure 4, it can be concluded that the (TG)_12_T_5_/(TG)_11_T_9_ genotype is the better fit for the observed chromatograms.

### 2.3. Evaluation of CFTR-TIPS

We assembled a cohort of 835 clinical samples tested at Mayo Clinic between September 2022 and December 2023. These samples underwent Sanger sequencing of the *CFTR* intron 9–exon 10 junction region. Subsequently, a clinically validated manual workflow was used to determine the (TG)_m_T_n_ genotype. The distribution of genotypes in these samples is shown in Table 1.

We then analyzed this cohort using *CFTR*-TIPS. *CFTR*-TIPS was able to successfully infer the (TG)_m_T_n_ genotype for 832 (99.6%) of the 835 samples. For the three failed samples, *CFTR*-TIPS encountered errors and was unable to infer the (TG)_m_T_n_ genotype. The error message, in lieu of the peak patterns, was displayed in the output section of the tool (Figure 3B). Two of the failed samples were due to an inability of the tool to detect the (TG)_m_T_n_ tract, and one was because *CFTR*-TIPS was unable to find a matching (TG)_m_T_n_ genotype.

**Figure 5:**
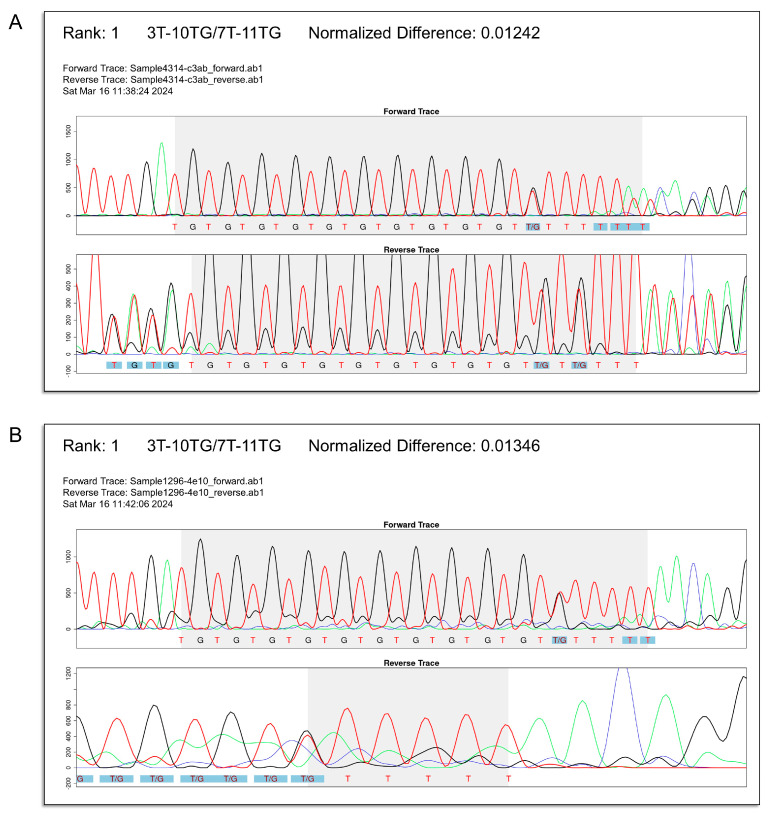
*CFTR*-TIPS GUI output for the two samples with discordant results. In these two samples, the first-ranked genotype inferred by *CFTR*-TIPS and the genotype determined by the clinically validated manual workflow were discordant. This figure shows the output section of the *CFTR*-TIPS GUI displaying the first-ranked (TG)_m_T_n_ genotype. (**A**) A T_7_/T_7_ sample misclassified as (TG)_10_T_3_ /(TG)_11_T_7_. *CFTR*-TIPS was confounded by the presence of a heterozygous 4 bp duplication in the same Sanger amplicon, as indicated by the overlapping peaks with the 3′ end of the poly-T tract in the reverse chromatogram. (**B**) A (TG)_11_T_5_/T_7_ sample misclassified as (TG)_11_T_5_/(TG)_10_T_6_. *CFTR*-TIPS was unable to fully detect the (TG)_m_T_n_ tract in the reverse chromatogram for this sample.

For the remaining 832 samples, we compared the first-ranked (TG)_m_T_n_ genotype inferred by *CFTR*-TIPS with that determined by manual review. Reassuringly, the results were concordant for 830 (99.8%) of the 832 samples. One discordant sample (manual: T_7_/T_7_; *CFTR*-TIPS: (TG)_11_T_7_/(TG)_10_T_3_, Figure 5A) had a 4 bp duplication in the same Sanger amplicon, which confounded *CFTR*-TIPS in detecting the (TG)_m_T_n_ tract. The other discordant sample (manual: (TG)_11_T_5_/T_7_; *CFTR*-TIPS: (TG)_11_T_5_/(TG)_10_T_6_, Figure 5B) was caused by the inability of *CFTR*-TIPS to fully detect the (TG)_m_T_n_ tract in the reverse Sanger chromatogram. Notably, there were no misclassifications of clinically significant results, i.e., (TG)_12_T_5_ or (TG)_13_T_5_.

In addition, while the time burden was not formally assessed, *CFTR*-TIPS generally took less than 10 s per sample. Overall, using 835 samples with diverse (TG)_m_T_n_ genotypes, we demonstrated that *CFTR*-TIPS facilitates accurate, rapid, and user-friendly inference of the (TG)_m_T_n_ genotype of the *CFTR* gene.

## 3. Discussion

### 3.1. Our Findings Support the ACMG Recommendations

The ACMG recently recommended reporting the (TG)_m_ size when T_5_ is detected [16,17]. Our findings support these recommendations. In our clinical laboratory, we perform a *CFTR* genotyping assay for carrier screening and testing of symptomatic individuals. This assay automatically reflexes to the Sanger sequencing-based (TG)_m_T_n_ genotype analysis when a T_5_ allele is detected. Thus, the distribution of (TG)_m_ size of the T_5_ alleles within our cohort provides a largely unbiased representation of the population that underwent *CFTR* variant testing. 

Among the 803 T_5_ alleles identified in our cohort (out of 1670 alleles tested), only 203 (25.3%) were the clinically significant (TG)_12_T_5_ or (TG)_13_T_5_ allele. This proportion is largely in line with previous estimates [9]. Our finding suggests that the vast majority of T_5_ alleles are clinically benign, highlighting the necessity of determining the (TG)_m_ size for accurate risk stratification of T_5_ alleles. Thus, incorporating (TG)_m_ size analysis into *CFTR* variant testing workflows not only aligns with the ACMG recommendations but also substantially improves the clinical utility of the assay.

### 3.2. Limitations of CFTR-TIPS

The two discordant samples reveal limitations of *CFTR*-TIPS. First, *CFTR*-TIPS is not suitable for samples in which a heterozygous insertion, duplication, or deletion variant is suspected in the same Sanger amplicon. This can be recognized by overlapping peaks with the 5′ end of the TG tract in the forward chromatogram or with the 3′ end of the poly-T tract in the reverse chromatogram (as shown in Figure 5A). Second, when the GUI indicates that *CFTR*-TIPS failed to fully detect the (TG)_m_T_n_ tract in the forward and/or reverse chromatogram (as shown in Figure 5B), we recommend discarding the results. In both scenarios, the goodness-of-fit calculations may be confounded, leading to erroneous results. Fortunately, samples that fall within both limitations can be easily recognized and discarded when the *CFTR*-TIPS GUI is reviewed. 

### 3.3. Suggested Applications and Benefits of CFTR-TIPS

NGS is increasingly used in daily practice for detecting mutations in the *CFTR* gene, particularly for patients with suspected CF or *CFTR*-related disorders. As shown in Figure 1D, the (TG)_m_T_n_ genotypes can be readily resolved using NGS-based assays. Nonetheless, the ACOG recommends against full-gene sequencing for routine CF carrier screening [8]. As a result, CF carrier screening tests may be performed using targeted genotyping platforms (e.g., genotyping microarray, MALDI-TOF mass spectrometry, multiplex PCR) instead of NGS. In addition, due to cost considerations, targeted mutation panels may remain the first-line test for suspected affected individuals. As a result, many laboratories, including those in North America and Europe, continue to offer these panels.

In our laboratory, the test volume of the genotyping microarray-based *CFTR* mutation panel in 2023 was more than ten times that of the NGS-based *CFTR* full-gene sequencing assay. Since targeted genotyping typically cannot reliably determine the TG repeat size, a supplementary method (e.g., Sanger sequencing of the (TG)_m_T_n_ tract region) is needed to adhere to the ACMG recommendations. Moreover, clinical implementation and validation of NGS require significant capital investments and technical expertise, which may be inaccessible in resource-limited settings. Therefore, we are hopeful that our tool, used in conjunction with Sanger sequencing-based methods, will become and remain an integral part of *CFTR* molecular diagnostics.

In research and/or resource-limited clinical laboratory settings, *CFTR*-TIPS may be operated in the fully automated mode. This is because of the very high accuracy of the tool (99.8% in our evaluation) even without manual review. Nonetheless, when possible, a cursory manual review of the *CFTR*-TIPS GUI is recommended. In our study, the two discordant samples were easily identified during a manual review, resulting in 100% accuracy. In addition, in non-resource-limited settings, *CFTR*-TIPS may be used to assist review and/or confirm results by laboratory technologists, leading to improved accuracy and reduced reviewer time burden, particularly for rare (TG)_m_T_n_ genotypes. 

The development of *CFTR*-TIPS offers significant benefits for patients. One of the main advantages of *CFTR*-TIPS is its high accuracy in determining (TG)_m_T_n_ genotypes, even for rare alleles such as T_6_, T_8_, and T_11_ and in the fully automated mode. Since only T_5_ alleles in combination with longer TG repeats are clinically significant, the high accuracy of *CFTR*-TIPS is crucial for providing patients with precise variant classification (i.e., pathogenic versus benign) and clinical counseling.

In addition, *CFTR*-TIPS is designed to integrate seamlessly into routine diagnostic workflows. Its user-friendly GUI allow laboratory technologists to quickly learn and efficiently use the software. This reduces the risk of errors and shortens the turnaround time, compared with manual review of the Sanger chromatograms. Taken together, these features of *CFTR*-TIPS ensure that patients receive timely and reliable diagnostic information, consequently improving the quality of care and supporting better clinical decision making.

## 4. Materials and Methods

### 4.1. Software Development, Implementation, Testing, and Availability

*CFTR*-TIPS was implemented using the R programming language (https://www.r-project.org/, accessed on 2 August 2024, version 4.3.1). The shiny package (version 1.7.5.1) was used to construct the graphical user interface. Other required R packages included bslib (version 0.5.1), tools (version 4.3.1), and sangerseqR (version 1.38.0) [21], as well as their dependencies. *CFTR*-TIPS is compatible with Windows, macOS, and Linux operating systems with the RStudio software installed. *CFTR*-TIPS was primarily tested on a computer with an Intel i5-12600 CPU and 16 GB RAM, running Windows 10 Enterprise, RStudio build 524 (version 2023.06.1), and Google Chrome version 122.0.6261.122.

The creation of *CFTR*-TIPS incorporated several key considerations to ensure its accuracy, reliability, and compatibility. We developed *CFTR*-TIPS using the RStudio/Shiny platform due to its free and open-source availability, as well as its broad compatibility across operating systems. Central to *CFTR*-TIPS is an algorithm designed to accurately identify the *CFTR* (TG)_m_T_n_ tract by detecting the 15 bp 5′ and 3′ flanking sequences of the tract. The flanking sequence length was carefully chosen to balance sequence uniqueness (i.e., ensuring that they are not found elsewhere in or near the *CFTR* gene) and maximum compatibility with various PCR primer designs. The sangerseqR package was selected to process the input Sanger chromatograms. Specifically, it converts the input .ab1 files, which are not directly readable by R, into R-compatible data structures. Additionally, sangerseqR performs base/peak calling, which is essential for *CFTR*-TIPS to accurately detect the (TG)_m_T_n_ tract and perform goodness-of-fit calculations.

The informatics of *CFTR*-TIPS was also designed to reliably handle the diversity of (TG)_m_T_n_ genotypes in the human population and the technical variations in the quality of input Sanger chromatograms. In particular, the *CFTR*-TIPS algorithm can recognize individuals heterozygous for alleles with different (TG)_m_T_n_ tract lengths, ensuring that overlapping peaks with the (TG)_m_T_n_ tract flanking regions do not interfere with the goodness-of-fit calculations. Through the “Minimum informative Sanger trace signal” parameter in the GUI, users can optionally adjust *CFTR*-TIPS to better accommodate the specific signal and noise levels of their Sanger chromatograms by discarding signals below the threshold as noise.

Moreover, *CFTR*-TIPS was created with user-friendliness as a priority. The software has robust error-handling features to guide users through common issues. For example, it provides clear error messages when the (TG)_m_T_n_ tract is not detected or when no combinations of TG and T repeat sizes in the user-defined search space match the uploaded data. Additionally, the *CFTR*-TIPS GUI offers instructions and demo files to assist users in troubleshooting. This user-centric approach enhances the overall usability of the software. *CFTR*-TIPS underwent rigorous testing to ensure its accuracy and compatibility. We evaluated *CFTR*-TIPS, as presented in this manuscript, using a wide range of Sanger chromatograms, encompassing various (TG)_m_T_n_ genotypes, laboratory instruments, and technologists. Additionally, feedback from initial users was incorporated to improve the GUI design. These comprehensive testing efforts ensure that *CFTR*-TIPS is a dependable tool for *CFTR* molecular diagnostics.

A preview of *CFTR*-TIPS is available at https://qd29.shinyapps.io/cftr-tips/ (accessed on 2 August 2024). Source code is available at https://github.com/qd29/cftr-tips/ (accessed on 2 August 2024). Compared with locally deployed versions (using the source codes), the preview version has several limitations. First, the preview version may be substantially slower and may occasionally encounter errors not attributable to *CFTR*-TIPS (such as HTTP 504 gateway timeout). Second, the preview version only allows one sample to be analyzed at a time. It is necessary to refresh the *CFTR*-TIPS webpage before analyzing another sample. Local versions do not have this restriction. Finally, while the preview version does not retain any user data, it is not hosted on a Health Insurance Portability and Accountability Act (HIPAA)-compliant server; thus, we recommend only uploading data from de-identified and/or research samples.

### 4.2. Goodness-of-Fit Calculation for Possible (TG)_m_T_n_ Genotypes

We quantified goodness of fit using a normalized difference score (D), as follows. This score was based on squared Euclidean distance, with lower scores denoting better goodness of fit.

(1) $$D=\frac{\sum_{i=1}^{n_{F}+n_{R}}{\left(O_{Gi}-E_{Gi}\right)}^{2}+{\left(O_{Ti}-E_{Ti}\right)}^{2}}{{2\times (n}_{F}+n_{R})}+\frac{100-m_{F}-m_{R}}{1\times 10^{5}}$$

Here, $n_{F}$ and $n_{R}$ denote the length of the *observed* (TG)_m_T_n_ tract in the forward and reverse chromatograms, respectively. $m_{F}$ and $m_{R}$ denote the length of the *expected* tract in the forward and reverse chromatograms, respectively. $O_{Gi}$ and $O_{Ti}$ denote the *observed* relative signal intensity (i.e., signal intensity of a given nucleotide divided by total signal intensity) of guanine and thymine at position i, respectively. $E_{Gi}$ and $E_{Ti}$ denote the *expected* relative signal intensity of guanine and thymine at position i, respectively. If thymine was expected at this position, $E_{Ti}$ was set to 1 and $E_{Gi}$ to 0, and vice versa. When both thymine and guanine were expected, both $E_{Ti}$ and $E_{Gi}$ were set to 0.5.

### 4.3. Cohort for Evaluation of CFTR-TIPS

To evaluate *CFTR*-TIPS, we assembled a cohort of 835 clinical samples tested at the CLIA-certified, CAP-accredited Molecular Technologies Laboratory in the Department of Laboratory Medicine and Pathology, Mayo Clinic (Rochester, MN, USA). Most samples were sequenced as a reflex for non-T_7_ (particularly T_5_) alleles detected by a *CFTR* genotyping assay.

Bidirectional Sanger sequencing was performed for the *CFTR* intron 9–exon 10 junction region; subsequently, the (TG)_m_T_n_ genotype was determined by a clinically validated workflow. This workflow was based on manual review of the data by experienced technologists. Due to lack of clinical significance, the manual workflow did not report (TG)_m_ status for non-T_5_ alleles. See below for technical details of PCR and Sanger sequencing. 

The genotype distribution of these samples is shown in Table 1. In addition to the T_5_, T_7_, and T_9_ alleles, the T_6_, T_8_, and T_11_ alleles were also observed in our cohort.

### 4.4. PCR and Sanger Sequencing

The PCR primers for the *CFTR* intron 9–exon 10 junction region were as follows: forward, 5′-CCATGTGCTTTTCAAACTAATTG-3′; reverse, 5′-CCAAAAATACCTTCCAGCACTACA-3′. Universal sequencing adapter sequences were included at the end of both primers. The expected amplicon size was 427 bp. A 10 μL PCR reaction contained 5.72 μL PCR-grade water, 2.00 μL KAPA2G buffer A, 0.20 μL KAPA2G dNTP mix, 0.08 μL KAPA2G enzyme, 1.50 μL primer mix (concentration of each primer: 1.25 μM), and 0.50 μL patient DNA (acceptable concentration: 80–250 ng/μL). 

PCR was performed on Applied Biosystems Veriti thermal cyclers with the following program: 3 min at 98 °C, followed by 15 cycles of 30 s at 95 °C, 30 s at 64.5 °C (−0.5 °C per cycle at a 50% ramp rate), and 60 s at 72 °C, followed by 20 cycles of 30 s at 95 °C, 30 s at 58 °C, and 60 s at 72 °C, followed by 10 min at 72 °C, and finally hold at 4°C. After PCR, the amplification products were purified using AMPure XP reagents. Sanger sequencing reactions were performed using universal sequencing primers. Applied Biosystems 3730xl DNA analyzers were used for capillary electrophoresis. 

## 5. Conclusions

In this study, we described and benchmarked *CFTR*-TIPS, a software tool that infers the *CFTR* (TG)_m_T_n_ genotype from Sanger chromatograms. When operated fully automatically (i.e., when the first-ranked genotype inferred by *CFTR*-TIPS was accepted without manual review), it achieved 99.8% concordance (830 out of 832 samples) with the clinically validated manual workflow. In conjunction with cost-effective Sanger sequencing, we are hopeful that *CFTR*-TIPS will facilitate access to *CFTR* (TG)_m_T_n_ genotype analysis for more patients, in accordance with the ACMG recommendations.

## Figures and Tables

**Table 1:** Genotype distribution of the cohort used for evaluation of *CFTR*-TIPS.

Genotype	Total Samples	Concordant (%)	Discordant (%)	Failed (%)
(TG)_11_T_5_/(TG)_11_T_5_	12	12 (100.0%)	0 (0.0%)	0 (0.0%)
(TG)_11_T_5_/(TG)_12_T_5_	5	5 (100.0%)	0 (0.0%)	0 (0.0%)
(TG)_11_T_5_/T_7_	495	493 (99.6%)	1 (0.2%) ^1^	1 (0.2%)
(TG)_11_T_5_/T_9_	76	76 (100.0%)	0 (0.0%)	0 (0.0%)
(TG)_12_T_5_/T_7_	147	146 (99.3%)	0 (0.0%)	1 (0.7%)
(TG)_12_T_5_/T_9_	25	25 (100.0%)	0 (0.0%)	0 (0.0%)
(TG)_13_T_5_/T_7_	19	19 (100.0%)	0 (0.0%)	0 (0.0%)
(TG)_13_T_5_/T_9_	2	2 (100.0%)	0 (0.0%)	0 (0.0%)
T_7_/T_7_	21	19 (90.4%)	1 (4.8%) ^2^	1 (4.8%)
T_7_/T_9_	12	12 (100.0%)	0 (0.0%)	0 (0.0%)
T_7_/T_11_	2	2 (100.0%)	0 (0.0%)	0 (0.0%)
T_9_/T_9_	12	12 (100.0%)	0 (0.0%)	0 (0.0%)
Other ^3^	7	7 (100.0%)	0 (0.0%)	0 (0.0%)
Total	835	830	2	3

^1^ Misclassified by *CFTR*-TIPS as (TG)_11_T_5_/(TG)_10_T_6_. ^2^ Misclassified by *CFTR*-TIPS as (TG)_10_T_3_/(TG)_11_T_7_. ^3^ Contains one sample each of (TG)_12_T_5_/(TG)_12_T_5_, (TG)_12_T_5_/(TG)_13_T_5_, (TG)_12_T_5_/T_6_, T_6_/T_7_, T_6_/T_9_, T_8_/T_9_, and T_9_/T_11_.

## Data Availability

A preview of *CFTR*-TIPS is available at https://qd29.shinyapps.io/cftr-tips/ (accessed on 2 August 2024). Source code is available on GitHub at https://github.com/qd29/cftr-tips/ (accessed on 2 August 2024).
